# 国产硼替佐米联合来那度胺、地塞米松治疗初治多发性骨髓瘤的多中心、前瞻性、Ⅱ期、单臂研究

**DOI:** 10.3760/cma.j.cn121090-20231217-00318

**Published:** 2024-06

**Authors:** 琳娜 解, 鑫 王, 强 贺, 慧 王, 骥 马, 海燕 张, 南 刘, 贵涛 接, 太武 肖, 颢 张, 海国 张, 增军 李, 立杰 邢

**Affiliations:** 1 山东第一医科大学附属肿瘤医院，山东省肿瘤医院淋巴血液病区，济南 250000 Department of Lymphohematology, Cancer Hospital of Shandong First Medical University, Shandong Cancer Hospital, Jinan 250000, China; 2 临沂市人民医院血液科，临沂 276003 Department of Hematology, Linyi City People's Hospital, Linyi 276003, China; 3 菏泽市立医院血液科，菏泽 274031 Department of Hematology, Heze City Hospital, Heze 274031, China; 4 临沂市中心医院血液科，临沂 276400 Department of Hematology, Linyi Central Hospital, Linyi 276400, China; 5 聊城市人民医院血液科，聊城 252000 Department of Hematology, Liaocheng City People's Hospital, Liaocheng 252000, China; 6 济宁医学院附属医院血液科，济宁 272100 Department of Hematology, Jining Medical University Affiliated Hospital, Jining 272100, China; 7 济宁市第一人民医院血液科，济宁 272011 Department of Hematology, Jining First People's Hospital, Jining 272011, China

**Keywords:** 多发性骨髓瘤, 硼替佐米, 来那度胺, Multiple myeloma, Bortezomib, Lenalidomide

## Abstract

**目的:**

探索国产硼替佐米联合来那度胺及地塞米松治疗初治多发性骨髓瘤（NDMM）的疗效和安全性。

**方法:**

此项多中心、前瞻性、单臂临床研究纳入2019年12月至2022年1月7所医院收治的126例NDMM患者，均接受国产硼替佐米联合来那度胺、地塞米松方案（BLD方案）治疗，分析其疗效、预后影响因素及安全性。

**结果:**

126例NDMM患者中，118例患者完成4个周期治疗，其中118例的总体反应率（ORR）为93.22％（110/118），≥非常好的部分缓解（VGPR）率为68.64％（81/118）。114例患者完成至少8个周期治疗，ORR为92.98％（106/114），≥VGPR率为77.19％（88/114）。18例在完成6～8个周期BLD方案治疗后进行自体造血干细胞移植，ORR为100％（18/18），≥VGPR率为88.9％（16/18）。治疗疗程增加与患者≥VGPR率升高呈正相关，且分期和年龄等因素对疗效无显著影响。单因素分析显示，R2-ISS分期Ⅲ期/Ⅳ期、血钙>2.27 mmol/L、6个周期疗效未达VGPR是无进展生存（PFS）的不良预后因素（*P*均<0.05），6个周期未达VGPR是OS的不良预后因素（*P*<0.001）。多因素分析显示，6个周期未达VGPR是PFS较差的独立预后因素（*P*＝0.002）。血液学不良反应发生率为16.7％（19/114），非血液学不良反应主要为轻度至中度，未发现明显心脏及肾脏不良反应。

**结论:**

BLD方案治疗NDMM效果显著，具有高危遗传学特征的患者仍可获得较高的≥VGPR率，且整体安全性良好。

## Abstract (en)

**Objective:**

To explore the efficacy and safety of domestic bortezomib in combination with lenalidomide and dexamethasone in the treatment of newly diagnosed multiple myeloma（NDMM）.

**Methods:**

This multicenter, prospective, single-arm clinical study included 126 patients with NDMM admitted to seven hospitals between December 2019 and January 2022. All patients received domestic bortezomib in combination with lenalidomide and dexamethasone（BLD regimen）, and the efficacy, prognostic factors, and safety were analyzed.

**Results:**

Among the 126 patients with NDMM, 118 completed four cycles of treatment, with an overall response rate（ORR）of 93.22％（110/118）and a ≥very good partial response（VGPR）rate of 68.64％（81/118）. Ultimately, 114 patients completed at least eight cycles of treatment, with an ORR of 92.98％（106/114）and a ≥VGPR rate of 77.19％（88/114）. Eighteen patients underwent autologous hematopoietic stem cell transplantation after completing 6–8 cycles of the BLD regimen, with an ORR of 100％（18/18）and a ≥VGPR rate of 88.9％（16/18）. The proportion of patients achieving ≥VGPR increased with the treatment duration, and factors such as staging and age did not significantly affect efficacy. Single-factor analysis showed that R2-ISS stage Ⅲ/Ⅳ, blood calcium >2.27 mmol/L, and failure to achieve VGPR after six cycles were adverse prognostic factors for progression-free survival（PFS）（*P*<0.05）, whereas failure to achieve VGPR after six cycles was an adverse prognostic factor for overall survival（OS）（*P*<0.001）. Multifactor analysis demonstrated that failure to achieve VGPR after six cycles is an independent adverse prognostic factor for PFS（*P*＝0.002）. The incidence of hematologic adverse reactions was 16.7％（19/114）, and nonhematologic adverse reactions were mainly mild to moderate, with no significant cardiac or renal adverse reactions observed.

**Conclusion:**

The BLD regimen is effective in treating NDMM, in which patients with high-risk genetic features are still achieving a high ≥VGPR rate, and the overall safety is good.

多发性骨髓瘤（MM）是一种常见的血液系统恶性肿瘤[1]–[3]，其特征为骨髓中克隆性浆细胞异常增生，分泌单克隆免疫球蛋白或其片段（M蛋白），并导致相应器官或组织损伤，临床常表现为骨痛、贫血、肾功能不全、感染、高钙血症等。MM多发于老年人，治疗疗程长，且目前仍无法治愈。随着蛋白酶体抑制剂、免疫调节剂、CD38单克隆抗体等新药的应用，及造血干细胞移植、嵌合抗原受体T细胞等免疫治疗的开展，MM患者的生存得到了较大改善。目前第一代蛋白酶体抑制剂硼替佐米、来那度胺和地塞米松联合治疗是国内外指南推荐的一线方案。本研究分析了山东省7个中心114例初治MM（NDMM）患者接受国产硼替佐米联合来那度胺、地塞米松治疗的疗效及安全性。

## 病例与方法

1. 病例：本研究为前瞻性临床研究，纳入山东省肿瘤医院、临沂市人民医院、菏泽市立医院、临沂市中心医院、聊城市人民医院、济宁医学院附属医院及济宁市第一人民医院自2019年12月至2022年1月接受国产硼替佐米、来那度胺及地塞米松（BLD）方案化疗的NDMM患者，中位随访24个月。诊断参考中国多发性骨髓瘤诊治指南（2020年修订）[3]，荧光原位杂交（FISH）检测提示存在del（17p）、t（4;14）、t（14;16）定义为细胞遗传学高危，其余为标危。本研究获得山东省肿瘤医院医学伦理委员会批准，并注册临床研究（CChiCTR2000040808），患者均同意并签署知情同意书。

2. 治疗方案：患者均接受BLD方案，参考国际标准方案，具体为：硼替佐米1.3 mg/m^2^，第1、4、8、11天，皮下注射；来那度胺25 mg，第1～14天，口服；地塞米松20 mg，第1、2、4、5、8、9、11、12天，21 d为1个周期，根据患者血肌酐清除率调整来那度胺剂量，治疗期间口服阿司匹林肠溶片预防静脉血栓，阿昔洛韦预防病毒感染，双歧杆菌调节肠道菌群。

3. 近期疗效及不良反应评估：参考中国多发性骨髓瘤诊治指南（2020年修订）[3]进行疗效和微小残留病（MRD）评估，分为完全缓解（CR）、非常好的部分缓解（VGPR）、部分缓解（PR）、微小缓解（MR）、疾病稳定（SD）、疾病进展（PD）。总反应率（ORR）为CR率、VGPR率、PR率之和。根据美国国家癌症研究所常见不良反应评价标准（CTCAE）5.0版对不良反应进行评估并分级。

4. 随访：通过门诊、住院病历和电话进行随访。本研究的随访截止时间为2023年1月1日，中位随访时间为28.9（26.1～31.7）个月。

5. 统计学处理：采用SPSS 24.0软件进行统计学分析。连续性变量采用均值±标准差描述，分类变量采用中位数（范围）。组间差异分析采用非参数检验。采用Kaplan-Meier法进行生存分析，Cox回归进行多因素分析。*P*<0.05为差异有统计学意义。

## 结果

1. 病例：126例NDMM患者入组，经过2个周期BLD方案治疗后，1例截瘫患者治疗1个周期后未再返院，4例患者因治疗后疾病进展自动退组；121例患者接受了4个周期BLD方案治疗，期间2例患者因无法耐受不良反应退组；1例患者因疾病进展退组；4例患者因放弃治疗等原因退组。最终共114例患者完成至少8个周期治疗。

2. 临床资料：114例患者中男65例（57.1％），女49例（42.9％），中位年龄61（40～87）岁。年龄<60岁患者53例（46.5％），≥60岁患者61例（53.5％）。IgG型41例（36.0％），IgA型39例（34.2％），IgD型3例（2.6％），轻链型26例（22.8％），未分泌型5例（4.4％）。DS分期Ⅰ期10例（8.8％），Ⅱ期8例（7.0％），Ⅲ期96例（84.2％）。ISS分期Ⅰ期44例（36.8％），Ⅱ期39例（35.9％），Ⅲ期3例（27.3％）。对完成FISH检测的58例患者行R-ISS分期：Ⅰ期15例（25.9％），Ⅱ期30例（51.7％），Ⅲ期13例（22.4％）。R2-ISS分期：Ⅰ期13例（22.4％），Ⅱ期12例（20.6％），Ⅲ期28例（48.2％），Ⅳ期5例（8.6％）。58例完成FISH检测的患者细胞遗传学异常（CA）结果示：del（17p13）13例（22.4％），1q21扩增23例（39.7％），IgH重排29例（43.1％），RB1（13q14）缺失8例（13.8％），t（11;14）3例（5.2％），t（4;14）2例（3.4％）。1个CA 21例（36.2％），2个CA 12例（20.7％），3个CA 7例（12.1％），4个CA 2例（3.4％）。

3. 疗效：4个周期治疗后评估疗效，CR 38例（32.48％），VGPR 43例（36.75％），PR 29例（24.79％），ORR为94.02％（110/118），≥VGPR率为69.23％（81/118）。8个周期治疗后疗效评估，CR 55例（48.25％），VGPR 33例（28.95％），PR 18例（15.79％），ORR为92.98％（106/114），≥VGPR率为77.19％（88/114）。18例患者完成6～8个周期BLD方案治疗并进行自体造血干细胞移植，CR 11例（61.1％），VGPR 5例（27.8％），PR 2例（11.1％），ORR为100％（18/18），≥VGPR率为88.9％（16/18）。

4. 分期对疗效的影响：4个周期治疗后疗效评估显示，ISS分期Ⅰ～Ⅱ期和Ⅲ期患者≥VGPR率（70.7％对68.8％）、ORR（93.9％对96.9％）的差异均无统计学意义（*P*值分别为0.835和0.523）。R-ISS分期Ⅰ～Ⅱ期和Ⅲ期患者≥VGPR率（64.4％对84.6％）和ORR（93.3％对92.3％）的差异均无统计学意义（*P*值分别为0.166和0.845）。

5. CA对疗效的影响：对合并不同CA患者进行疗效评估。45例患者无17p−，13例伴17p−，ORR分别为93.3％（42/45）和92.3％（12/13），组间比较差异无统计学意义（*χ*^2^＝0.017，*P*＝0.898）。35例患者无1q21+，23例伴1q21+，ORR分别为91.4％（32/35）和95.7％（22/23），组间比较差异无统计学意义（*χ*^2^＝0.386，*P*＝0.535）。29例患者无IgH重排，29例伴IgH重排，ORR分别为93.1％（27/29）和89.7％（26/29），组间比较差异无统计学意义（*χ*^2^＝0.783，*P*＝0.376）。31例无13q14−，27例伴13q14−，ORR分别为90.3％（28/31）和96.3％（26/27），组间比较差异无统计学意义（*χ*^2^＝0.802，*P*＝0.370）。

6. 生存情况：患者治疗后中位无进展生存（PFS）时间未达到，2年PFS率77.5％。单因素分析显示，R2-ISS分期Ⅲ期/Ⅳ期、血钙>2.27 mmol/L、6个周期疗效未达VGPR是PFS的不良预后因素（*P*均<0.05）。将上述因素纳入多因素分析，结果显示，6个周期疗效未达VGPR是PFS的不良预后因素（*P*＝0.002）（表1）。

**表1:** 影响初治多发性骨髓瘤患者无进展生存的单因素及多因素分析

因素	单因素分析		多因素分析	
	*HR*（95%*CI*）	*P*值	*HR*（95%*CI*）	*P*值
年龄（≥60岁，<60岁）	1.77（0.76～4.10）	0.184		
性别（男，女）	1.22（0.56～2.67）	0.621		
HGB（≤100 g/L，>100 g/L）	0.60（0.27～1.30）	0.194		
肌酐（≤104 g/L，>104 g/L）	1.73（0.69～4.34）	0.242		
β_2_-微球蛋白（≤2 mg/L，>2 mg/L）	1.91（0.45～8.12）	0.379		
LDH（≤205 g/L，>205 g/L）	1.30（0.57～2.93）	0.533		
血钙（≤2.27 mmol/L，>2.27 mmol/L）	0.41（0.18～0.95）	0.038	1.30（0.38～4.45）	0.679
白蛋白（≤37.7 g/L，>37.7 g/L）	1.39（0.63～3.10）	0.418		
ISS分期Ⅲ期（是，否）	1.29（0.58～2.99）	0.553		
R-ISS分期Ⅲ期（是，否）^a^	2.24（0.66～7.67）	0.198		
R2-ISS分期Ⅲ期/Ⅳ期（是，否）^a^	8.14（1.04～63.62）	0.046	4.59（0.52～40.26）	0.169
伴17p−（是，否）^a^	2.83（0.75～10.66）	0.125		
伴1q21+（是，否）^a^	1.12（0.34～3.69）	0.849		
伴13q−（是，否）^a^	1.56（0.34～7.23）	0.571		
治疗6个周期未达VGPR（是，否）	29.77（9.99～88.73）	<0.001	7.87（2.17～28.48）	0.002

**注** ^a^例数为58例；VGPR：非常好的部分缓解

患者中位总生存（OS）时间未达到，单因素分析显示，6个周期未达VGPR是OS的不良预后因素（*P*<0.001）（表2）。

**表2:** 影响初治多发性骨髓瘤患者总生存的单因素分析

因素	单因素分析	
	*HR*（95%*CI*）	*P*值
年龄（≥60岁，<60岁）	1.37（0.50～3.77）	0.542
性别（男，女）	1.22（0.46～3.26）	0.689
HGB（≤100 g/L，>100 g/L）	0.44（0.16～1.18）	0.102
肌酐（≤104 g/L，>104 g/L）	2.61（0.91～7.53）	0.075
β_2_-微球蛋白（≤200 g/L，>200 g/L）	1.13（0.26～4.98）	0.870
LDH（≤205 g/L，>205 g/L）	1.89（0.70～5.08）	0.207
血钙（≤2.27 mmol/L，>2.27 mmol/L）	2.45（0.85～7.07）	0.096
白蛋白（≤37.7 g/L，>37.7 g/L）	1.49（0.54～4.09）	0.443
ISS分期Ⅲ期（是，否）	1.50（0.55～4.13）	0.433
R-ISS分期Ⅲ期（是，否）^a^	3.47（0.70～17.23）	0.128
R2-ISS分期Ⅲ期/Ⅳ期（是，否）^a^	3.47（0.40～29.83）	0.257
伴17p−（是，否）^a^	1.39（0.16～11.91）	0.766
伴1q21+（是，否）^a^	0.63（0.28～8.76）	0.600
伴13q−（是，否）^a^	3.83（0.69～21.35）	0.125
治疗6个周期未达VGPR（是，否）	33.05（7.44～146.78）	<0.001

**注** ^a^例数为58例；VGPR：非常好的部分缓解

7. 不良事件：BLD方案所致的不良反应主要包括周围神经病变、乏力、消化道症状、血液学不良反应及感染等，血液学不良反应发生率为16.7％，常见的非血液学不良反应包括乏力（30.7％）、周围神经病变（31.5％）、腹泻/腹胀/便秘（28.9％）及感染（18.4％），均为CTCAE分级1～2级，经对症治疗可改善。消化道症状多出现在第2～3个疗程，表现为腹泻、腹胀及便秘等，予对症治疗可改善。周围神经病变多出现在第3～4个疗程，为1～2级，患者表现为手足麻木，予对症治疗可缓解。由于采用阿昔洛韦预防病毒感染，患者在治疗早期出现带状疱疹比例不高，12例患者出现带状疱疹，多为治疗后期未按时服用抗病毒药物所致。未发现明显心脏及肾脏不良反应。

## 讨论

MM是一种异质性强，目前仍不可治愈的血液系统肿瘤，5年OS率约55％[4]，激素、免疫调节剂及蛋白酶体抑制剂组成的两药或三药联合方案被广泛应用于MM的诱导治疗。硼替佐米、来那度胺及地塞米松已成为目前公认的NDMM一线方案，ORR可达90％，且约1/3患者可获得CR，20％患者可达MRD阴性[5]–[8]。全球多项临床试验数据显示三药联合对NDMM患者的长期疗效和安全性[9]–[12]，但上述研究结果均为欧美国家数据，且国内很多临床研究所用药物为进口原研药物。MM患者通常需要长期治疗，很多患者经济负担加重，无力承担规范治疗费用，影响了MM患者的总体生存水平。目前国产硼替佐米及来那度胺上市且纳入医保，国产药物组合在中国患者中的安全性、有效性等亟待进一步了解。

数据显示，患者4个周期化疗后ORR为93.97％，且4个周期化疗后CR率达32.76％，化疗继续进行至8个周期时CR率提升至46.5％。6个周期治疗后≥VGPR率（75.4％对66.6％）、ORR（94.7％对83.4％）高于PETHEMA/GEM2012的Ⅲ期研究临床研究数据[13]。BLD方案治疗8个周期后≥VGPR率（76.3％对65.7％）、ORR（92.1％对85.1％）高于SWOG SO777的Ⅲ期临床研究数据[14]。本研究数据显示国产药物的疗效不劣于原研药物。

以染色体畸变为主的CA的多样性可能是MM高度异质性的主要原因。CA可分为原发性CA（pCA）和继发性CA（sCA）两类，分别驱动MM的发生和疾病进展[15]。pCA中的IgH重排［t（4;14）、t（14;16）、t（14;20）］和sCA中的染色体拷贝数异常［1q21扩增、del（17p）］等被定义为高危CA，均具有独立的预后价值[16]–[17]。本研究共有58例患者进行间期FISH检测，其中的CA为IgH重排（43.1％）、1q21扩增（39.7％）、17p13−（22.4％）及13q14−（13.8％）。原瑞凤等[18]的研究纳入1 015例NDMM患者，del（13q）/13q14−占46.4％，1q21扩增占46.1％，del（17p）占9.9％。另一项多中心研究中553例NDMM进行FISH检测，CA中以del（13q）最为常见（60.4％），其次为IgH重排（57.6％）、1q扩增（49.0％）和del（17p）（34.7％）[18]，我国NDMM患者的CA谱显示，基于CA的高危MM在NDMM患者中所占比例近58％[18]。本研究结果显示高危MM占36.2％（21/58），较文献报道数据略低，可能与样本量不够大有关，但1q21扩增比例与国内报道数据接近。有CA患者与无CA患者ORR的差异无统计学意义。

新药联合应用延长了NDMM患者的PFS时间和OS时间。单因素分析里R2-ISS分期显示了对PFS的不良影响，提示高危CA患者PFS差，但这些因素未对OS产生影响，提示BLD方案治疗可能克服了这种差异。患者治疗6个周期后未达到VGPR会影响患者的PFS，显示了治疗效果对PFS的影响。本研究数据显示，4个周期治疗后ORR 94.7％，6个周期治疗后≥VGPR率75.4％，显示BLD方案联合治疗可以使大部分患者达到较好的治疗效果。17p−是NDMM的高危因素，在本研究中，单因素分析未观察到17p−是PFS、OS的独立不良预后因素，可能与纳入样本量不足有关。近期研究显示，1q+在中国MM患者的诊断中相对常见[19]，与OS较差相关，是接受硼替佐米诱导治疗患者OS的独立不良预后因素，自体造血干细胞移植可以部分减少1q+对生存的不良影响[20]，但本研究显示，1q21+没有对PFS及OS产生影响，可能是由于本研究样本量有限，需要进一步扩大样本量。

在安全性方面，国产药物不良反应与进口药物不良反应相似，硼替佐米的不良反应主要为周围神经病变（31.5％），该结果与SUMMIT/CREST试验报道（35％）相似[21]，其次是乏力（30.7％）、腹泻及腹胀（28.9％）等。本研究无3级以上神经系统不良反应，考虑与硼替佐米皮下注射有关。来那度胺的主要不良反应为骨髓抑制、中性粒细胞减少及血栓，血液学不良反应多为1~2级，安全性数据总体与进口药物相似。

综上所述，本研究结果显示BLD方案总有效率高，不良反应可控，患者的长期生存率仍有待更长时间的随访及更大样本的临床研究证实。
